# Parallel functional differentiation of an invasive annual plant on two continents

**DOI:** 10.1093/aobpla/plz010

**Published:** 2019-03-07

**Authors:** Andrew M Latimer, Brooke S Jacobs, Ernesto Gianoli, Tina Heger, Cristian Salgado-Luarte

**Affiliations:** 1 Department of Plant Sciences, University of California, Davis, CA, USA; 2 California Department of Fish and Wildlife, Sacramento, CA, USA; 3 Departamento de Biología, Universidad de La Serena, La Serena, Casilla, Chile; 4 Departamento de Botánica, Universidad de Concepción, Concepción, Casilla, Chile; 5 Biodiversity Research/Botany, University of Potsdam, Potsdam, Germany; 6 Technical University of Munich, Restoration Ecology, Freising, Germany; 7 Berlin-Brandenburg Institute of Advanced Biodiversity Research (BBIB), Berlin, Germany

**Keywords:** *Erodium cicutarium*, flowering time, functional trait correlations, invasive species, life-history strategy, local adaptation, parallel evolution, phenotypic plasticity

## Abstract

Rapid local adaptation frequently occurs during the spread of invading species. It remains unclear, however, how consistent, and therefore potentially predictable, such patterns of local adaptation are. One approach to this question is to measure patterns of local differentiation in functional traits and plasticity levels in invasive species in multiple regions. Finding consistent patterns of local differentiation in replicate regions suggests that these patterns are adaptive. Further, this outcome indicates that the invading species likely responds predictably to selection along environmental gradients, even though standing genetic variation is likely to have been reduced during introduction. We studied local differentiation in the invasive annual plant *Erodium cicutarium* in two invaded regions, California and Chile. We collected seeds from across strong gradients in precipitation and temperature in Mediterranean-climate parts of the two regions (10 populations per region). We grew seeds from maternal families from these populations through two generations and exposed the second generation to contrasting levels of water and nutrient availability. We measured growth, flowering time and leaf functional traits across these treatments to obtain trait means and plasticity measures. We found strong differentiation among populations in all traits. Plants from drier environments flowered earlier, were less plastic in flowering time and reached greater size in all treatments. Correlations among traits within regions suggested a coordinated evolutionary response along environmental gradients associated with growing season length. There was little divergence in traits and trait intercorrelations *between* regions, but strongly parallel divergence in traits *within* regions. Similar, statistically consistent patterns of local trait differentiation across two regions suggest that local adaptation to environmental gradients has aided the spread of this invasive species, and that the formation of ecotypes in newly invaded environments has been relatively consistent and predictable.

## Teaser

Originally from Europe, *Erodium cicutarium* (redstem storksbill) spread across both North and South America. The plant grows in diverse habitats from deserts to mountaintops. Has it evolved to grow differently in different habitats during these invasions, and do such differences help it spread? By experimentally growing seeds from many sites in California and Chile, this study discovered strong local genetic differences: plants from dry areas ‘live fast and die young’, flowering weeks earlier than plants from wetter areas. Patterns of local differences among Chilean populations matched those in California, suggesting this species evolves in predictable ways as it spreads.

## Introduction

Broadly distributed species encompass populations spread across habitats that vary in their climatic, edaphic and biotic environmental characteristics. The success of broadly distributed species across a wide range of environmental conditions is, in part, determined by their ability to maintain fitness and positive population growth rates across the range of local environmental conditions encountered by individual populations (Alleaume-Benharira *et al.* 2006). Genetic variation and phenotypic plasticity determine the extent to which trait expression in a population matches local selective optima (Callaway *et al.* 2003; Hereford 2009; Lazaro-Nogal *et al.* 2015). Both mean trait values and levels of phenotypic plasticity can evolve in response to local selection regimes, enabling a broadly distributed species to maximize fitness and persist across a wider range of environmental conditions than could any single constituent population or ecotype (Chevin *et al.* 2010).

Widespread invasive species are characterized by range expansions following introduction to a new location and often associated with broad ecological distributions across their native and invaded ranges (Richardson *et al.* 2000). As a result, invasive species can provide useful case studies for understanding the roles of genetic differentiation and plasticity in the process of range expansion (Sexton *et al.* 2002; Parker *et al.* 2003; Molina-Montenegro *et al.* 2013). Studies of the response of invasive species to novel conditions may also shed light on how established species respond to novel environmental conditions, including climate change, through evolutionary and plastic responses (Moran and Alexander 2014; Chown *et al.* 2015; Hobbs *et al.* 2018). An important question in invasion biology is to what extent differentiation of local populations—in mean traits, plasticity or both—aids exotic invasive species in extending their distributions across varying environments, and how rapidly such differentiation occurs. Because colonization events that begin plant invasions can involve a relatively small number of seeds or plant fragments, rapid differentiation during invasion may be restricted in some cases by low standing genetic variation in the spreading populations (Matesanz *et al.* 2014; Balogh and Barrett 2016). A review found that for most invasive species, population differentiation in the invaded range is lower than in the native range, suggesting that introduced populations may not have had time to differentiate fully (Bossdorf *et al.* 2005). Recent studies have, however, documented many cases of rapid evolution in invaded ranges (Maron *et al.* 2004; Montague *et al.* 2008; Prentis *et al.* 2008; Colautti and Barrett 2013; Oduor *et al.* 2016; Blossey *et al.* 2017; Moran *et al.* 2017). It appears that rapid trait differentiation in invasive species is the rule rather than the exception, but it remains unclear whether trait differentiation in the invaded range is due to local selection or other factors, and how differentiation in mean traits relates patterns of plasticity (Maron *et al.* 2007; Murren and Dudash 2012). Evaluating how predictable these evolutionary responses are will require empirical data on among-population functional traits in species introduced in multiple regions, data that remain largely lacking (Colautti and Barrett 2013; Colautti and Lau 2015).

Adaptive genetic differentiation in mean trait expression has been widely demonstrated in the field (Linhart and Grant 1996; Leimu and Fischer 2008; Hereford 2009; Fournier-Level *et al.* 2011). Previous studies have found strong differences in flowering time and growth rate across climatic gradients in both annual (Franks *et al.* 2007) and perennial plants (Weber and Schmid 1998; Colautti and Barrett 2013); these differences imply that there is strong selection on annual plants to match their phenology to local climate seasonality. Adaptive phenotypic plasticity, the ability of genotypes to match trait expression and selective optima across environments, provides another important mechanism for a broadly distributed species to maximize fitness in response to variable selection pressures (Richards *et al.* 2006; Holt and Barfield 2011; Molina-Montenegro *et al.* 2018). Greater phenotypic plasticity is expected to be favoured in habitats where populations encounter variation in selection within or across generations as a result of temporal or spatial environmental variation (Van Kleunen and Fischer 2001; Alpert and Simms 2002; Gianoli 2004; Gianoli and Gonzalez-Teuber 2005; Baythavong 2011; Pratt and Mooney 2013; Lazaro-Nogal *et al.* 2015), while selection should tend to favour adaptive genetic differentiation among populations exposed to consistent differences in selection on average trait expression (Linhart and Grant 1996; Sultan and Spencer 2002; Leimu and Fischer 2008). Theory suggests that adaptive plasticity may enable persistence in novel environmental conditions and facilitate local adaptation (Chevin and Lande 2010; Chevin *et al.* 2010; Franks *et al.* 2014).

Since individual phenotypic traits are linked through organismal structure and function, they often evolve in a correlated way (Savage and Cavender-Bares 2011). Suites of functionally related traits are thus expected to co-vary in ways that reflect ecological trade-off axes such as maximum growth rate versus resource efficiency or rapid reproduction versus extended growth. It is also possible, however, that the patterns of correlations among traits could themselves be sensitive to the environment—for example, growth rate might be tightly coupled to flowering time in a dry environment, but these traits might be less strongly linked in a mesic environment (Pigliucci and Schlichting 1995; Gianoli 2003). To use an example of trait correlations relevant to this study, if local populations of an exotic plant experience selection for shorter lifespan in environments with short growing seasons, we expect many other traits to co-vary with growing season length and plant lifespan. For example, we expect to see associations between growing season length, growth rate and flowering time, as well as growth-related leaf traits. If an invading species is exposed to similar environmental clines in different invaded ranges, we would expect populations of the species to respond similarly in each new invaded region (assuming sufficient additive genetic variation is present), producing parallel evolution of suites of functional traits and therefore similar sets of ecotypes in each region, as has been found in some other plant and animal species (Maron *et al.* 2004; Leger and Rice 2007; Jones *et al.* 2012; Oduor *et al.* 2016).

*Erodium cicutarium* is a broadly distributed annual forb that has invaded a wide range of habitats on all continents except Antarctica, from its native range in Europe (Fiz-Palacios *et al.* 2010). In California, where the species was introduced around 300 years ago (Mensing and Byrne 1998), it occurs from the Mojave Desert in the south to Sierra Nevada foothills >500 km to the north. In Chile, 170 years ago it was already referred as extremely abundant throughout the Mediterranean-climate region (30–35°S), from the border with the Atacama Desert in the north to the transition with temperate forests in the south (Barnéoud 1845). Its introduction date in Chile might therefore be comparable to that in California, though it is possible that it was introduced more recently in Chile.

These parallel invaded ranges of *E. cicutarium* in Chile and California provide an opportunity to characterize differentiation in mean trait expression and phenotypic plasticity on two continents with similar climates (Di Castri 1973; Mooney 1977) and to relate potential trait differentiation to environmental gradients and to performance under contrasting conditions. This parallel has been exploited previously to test whether patterns of trait differentiation in the introduced range of *Eschscholzia californica* in Chile are parallel to those in the species’ native range in California; the study found the patterns were parallel, suggesting adaptive evolution in the invasive range (Leger and Rice 2007). Here, we study local differentiation of an exotic species that has been introduced into both of these similar but independent regions. This comparison of parallel invasions gives us a more direct view whether patterns of local adaptation and plasticity variation during invasions are consistent and therefore potentially predictable (Oduor *et al.* 2016). Observing parallel responses does not, of course, prove that differentiation is adaptive, but consistent patterns of differentiation along two parallel gradients suggest that differentiation has been driven by selection (Endler 1986) rather than other evolutionary processes, such as multiple invasions and genotype sorting or genetic drift (Keller *et al.* 2009; Zenni *et al.* 2014).

**Figure 1.**
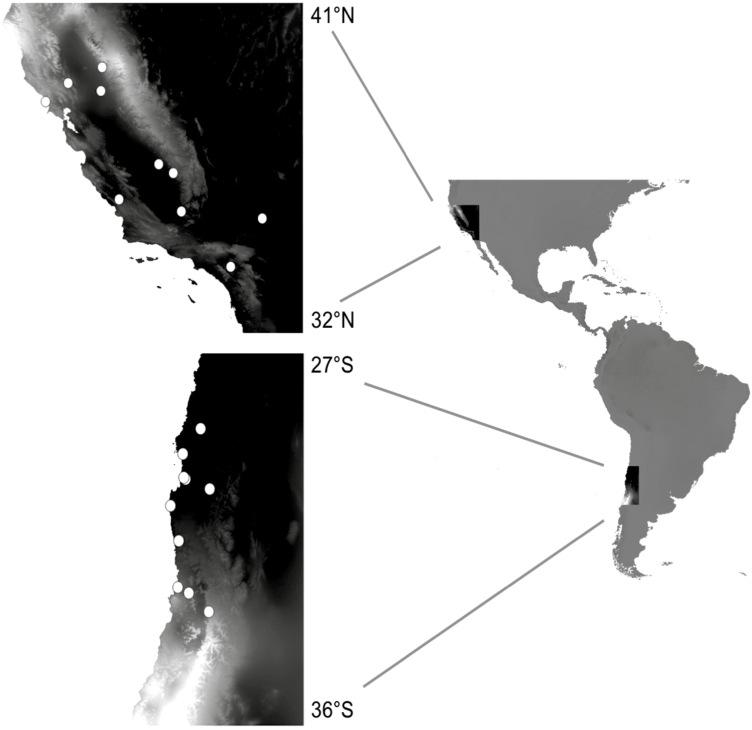
Locations of *Erodium cicutarium* source populations. White circles represent sites from which seeds were collected for use in greenhouse experiments. The background colour represents mean annual precipitation (lighter = wetter).

We collected seeds from maternal plants in populations across gradients in latitude, mean annual precipitation and precipitation seasonality (21 populations in California, 14 in Chile). For the greenhouse experiments, we selected 10 populations from each region that represented these gradients well and grew them out for one generation to equalize maternal environments (see Fig. 1). We conducted greenhouse experiments that included exposing a second generation of plants to contrasting water and nutrient treatments to characterize patterns of variation in mean trait expression and phenotypic plasticity. We also characterized climatic conditions and local edaphic and biotic environments in the source populations. We address the following questions: (i) Is there differentiation in flowering time, growth rate and leaf traits, and in the level of plasticity in these traits, among *E. cicutarium* populations from California and Chile? (ii) Are patterns of differentiation among populations in mean trait expression and plasticity associated with characteristics of the source sites? We hypothesize that: (a) flowering time and growth-related functional traits will vary along gradients in mean precipitation, which in these regions is strongly associated with productivity and growing season length (more precipitation leads to higher productivity over a longer growing season); and (b) higher plasticity will be associated with greater temporal and spatial environmental variation in availability of water and nutrients. (iii) What are the patterns of correlation among functional traits, and do these define a suite of traits whose variation along environmental gradients is consistent in the two invaded regions?

## Materials and Methods

### Field surveys

We selected populations of *E. cicutarium* along two north/south and two east/west transects in the Mediterranean-climate regions of California and Chile to capture a wide range of climatic conditions (Fig. 1; Table 1). Field surveys were conducted in spring 2010 in California (March) and Chile (September through November). At each site, 15–20 individuals with at least one mature seed were randomly selected within a 100 m^2^ area. Mature seeds were collected from each individual in the field and stored in envelopes for later greenhouse experiments. Soil samples were taken from the top 10 cm of soil at the locations of each mother plant from which seeds were collected. A subset of these soil samples was sent to A&L Agricultural Labs for basic soil analysis (Baythavong 2011). At the location of each maternal plant, we also measured general characteristics of the local plant community, including total plant cover, and percent cover of major functional types (grasses, forbs, shrubs). These multiple measurements per site allowed us to characterize both average edaphic and local biotic environmental conditions for each site and their degree of spatial variation within sites.

**Table 1.** Source-site environmental characteristics. MAP = mean annual precipitation; MAT = mean annual temperature; seasonality = standard deviation of monthly precipitation (January–December); CV = interannual coefficient of variation of precipitation (100 * [standard deviation/mean]; data from 1981 to 2010).

Region	Source population	Latitude (°N)	Longitude (°W)	MAP (mm)	MAT (°C)	Precip. seasonality	Precip. CV (%)
California	Motte	33.8045	117.2587	391	16.7	85	47
	Bodega Bay	38.3171	123.0692	1012	12.7	88	31
	Sweeney	35.1120	116.2677	69	21.4	46	54
	Sand Ridge	35.3082	118.7980	214	19.0	79	41
	Calcareous	35.6320	120.7530	477	13.7	92	41
	Lindcove	36.3584	119.0537	358	17.3	82	37
	Kearney	36.6027	119.5081	305	17.0	83	37
	Riverwood	38.6019	121.3272	475	16.2	88	34
	McLaughlin	38.8248	122.3474	803	14.1	90	35
	Foothills	39.2575	121.2889	971	15.4	84	32
Chile	Santiago	−33.4787	70.5169	509	13.6	96	50
	La Campana	−32.9804	71.1186	391	13.2	111	51
	Mantagua	−32.8298	71.4630	362	14.2	110	51
	Illapel	−31.5890	71.4352	184	16.6	98	57
	Fray Jorge	−30.6619	71.6827	150	13.6	123	58
	Alcohuaz	−30.2193	70.4935	76	9.9	120	51
	Cerro Grande	−29.9612	71.2162	88	14.5	112	69
	NeoHaus	−29.9139	71.2750	79	15.2	101	69
	Carrizal Bajo	−29.2917	71.3058	38	17.6	108	63
	Vallenar	−28.6288	70.7682	54	15.7	97	64

### Greenhouse experiments

Two brown, filled seeds per maternal family were randomly selected from each field-collected maternal family. Seeds were physically scarified to break dormancy and planted into pots using the same protocol as in previous *E. cicutarium* greenhouse experiments conducted by Baythavong (2011) to minimize differences in maternal environmental effects among families. Seeds produced by these first-generation plants were used in a second-generation greenhouse experiment.

For each region (California and Chile), we used 10 populations from sites that spanned similar ranges of latitude and precipitation (Table 1). Ten maternal families were randomly selected for each population from the set of families included in the first greenhouse generation. Eight brown, filled seeds per family were physically scarified and planted in October, using the same methods described for the first greenhouse generation (8 seeds per family, 10 families per population, 20 populations = 1600 total seeds). After emergence, two seedlings per family were placed into random locations within racks for each of the four treatment types (see below). Within a week after emergence, 147 seedlings were killed by herbivores (fly larvae) under the mist benches; this mortality was spread evenly across sites and source regions with no clear pattern of susceptibility or resistance. After this mortality, sample size ranged from 68 to 76 plants per site (of the initial 80). As a result, some families were represented by only one seedling in each treatment. One population (Bodega Bay, California) experienced anomalously high mortality during the experiment, resulting in a small sample size for estimating treatment effects and a highly unbalanced design when this population was included in experiment-wide analyses. We therefore removed this population from the analysis, leaving 19 populations (9 from California, 10 from Chile).

Seedlings were top-watered with fertilizer water for 1 week before treatments were imposed. After the first week, racks were randomly assigned to one of four treatments: high and low water, and high and low nutrients. Because fertilizer was delivered with the water, we did not cross nutrient and water treatments in the experiment, but instead conducted separate contrasts for water and nutrients. For the water treatments, we held nutrient concentrations in the water constant and varied the amount of water. For the nutrient treatments, we held the amount of water constant and varied the nutrient concentration.

High-water treatment racks were top-watered three times per week with 1 gallon of fertigated water throughout the experiment. Low-water treatment racks received a different watering schedule determined by measurements of percent water content of individual pots. Two individuals per low-treatment rack were randomly selected and weighed every other day to calculate percent water content. When the average water content reached 15 % averaged across ‘low-water’ pots, each rack was top-watered with 1 gallon of water. High- and low-nutrient treatment racks were top-watered three times per week with 1 gallon of fertigated water, with the level of nutrients controlled by the concentration in the fertigated water. Water for the low-nutrient plants was diluted 16 times compared to the water for the high-nutrient plants. High- and low-water treatment racks were watered with fertigated water diluted three times compared to the high-nutrient plants, and were also watered with 1 gallon of water at each watering time. Throughout the entire experiment, racks were rotated two times per week to minimize the effect of microenvironmental variation within the greenhouse on trait expression.

Surveys of flowering were conducted every other day until the experiment was terminated in late May (~7.5 months after seeds were sown). The number of days from planting to the date at which the first flower opened was recorded as ‘flowering time’. At the end of the experiment, the longest stem of each plant was measured to assess plant height. Specific leaf area (SLA) was calculated as the ratio of leaf area (mm^2^) to leaf weight (mg). A single, fully expanded leaf was randomly selected from each individual. The leaf was immediately pressed beneath glass and photographed. This leaf was then dried at 60 °C to a constant weight. Image J (http://rsbweb.nih.gov/ij/) was used to measure leaf area digitally, using hand tracing to deal with residual shadows in the images.

### Statistical analysis

#### Variation among populations in mean trait expression and plasticity.

All analyses were done using R version 3.4.3 (R Core Team 2018). We summarized the functional trait response variables (flowering time, stem length, SLA and leaf area) to family means within each treatment **[see****Supporting Information—Dataset S1****]**. To descriptively partition variance, we first used ANOVA to determine the amount of mean trait variation associated with population of origin, with greenhouse treatment, and the interaction of population of origin and treatment. To test for significant differences in traits between treatments, regions (California vs. Chile) and source populations within regions, we fitted linear mixed models. In these models, family mean trait values were the response variables, and we included the fixed effect of treatment (high water, low water, high nutrients or low nutrients) plus random intercepts for source population nested within region. Stem length, SLA and leaf area were log-transformed before analysis to homogenize residual variance.

We used a model comparison approach to test for significant differences in traits among regions (Chile and California) by using likelihood ratio tests to compare linear mixed models containing region as a random effect to null models containing no explanatory variables. We similarly tested for significant variation in traits among source populations by comparing models that included only treatment (fixed effect) and region (random effect) to models including these variables plus source population random effects nested within region. Finally, we tested for significant differences among treatments by comparing models that included treatment (fixed effect) and nested population and region random effects with models containing only population and region random effects.

We tested for variation in trait plasticity among regions and among populations in the same way, again using linear mixed models. Plasticity was calculated for each family as the absolute difference in family mean trait values between the high-resource greenhouse treatment (high water or high nutrients) and the corresponding low-resource treatment (low water or low nutrients), divided by the family mean across both treatments (Davidson *et al.* 2011).

We tested the association of the family mean plasticity values with region and population, again using likelihood ratio tests as described above. Because the plasticity response variables are a standardized difference between family means from two contrasting treatments (high vs. low water; high vs. low nutrients), the significance of treatment could not be evaluated for plasticity. We adjusted the sets of *P*-values from these tests for multiple comparisons by using the method of Benjamini and Hochberg to control the false discovery rate to <5 % (Benjamini and Hochberg 1995).

#### Association between population-level trait variation and source-site environmental variables.

To characterize climatic conditions of the source populations, mean annual temperature, mean annual precipitation and seasonality in precipitation (standard deviation of average monthly precipitation values from January to December) were obtained from the WorldClim online database (http://www.worldclim.org/). In addition, temporal variation in precipitation was calculated as the coefficient of variation of mean annual precipitation over 30 years, using monthly global interpolated precipitation data for 1981–2010 downloaded from the Climatic Research Unit of the University of East Anglia (Hulme 1992). We measured characteristics of the soil and the plant community at the locations of at 10 randomly selected *Erodium* maternal plants per source population. Because we did not have a clear hypothesis about which soil properties would likely influence our functional traits, other than general soil fertility, we used a principal component analysis on the standardized values of soil nutrients. The soil properties measured included total N, P and K, as well as pH and the micronutrients Mg, Ca, Na and S, as well as estimates of total organic matter and cation exchange capacity. The first principal component from this analysis accounted for 38 % of the variation in the soil data and was heavily weighted on calcium and magnesium content as well as cation exchange capacity and soil organic matter. Measurements of grass cover and total plant cover were strongly correlated (ρ = 0.76), so we chose to include only one of these two variables, total plant cover, in the analysis. We summarized the soil principal component and total plant cover to a mean value and a coefficient of variation for each population. Because there was only one value per population for the source-site environmental variables, we also summarized the trait response variables to population means for this analysis.

To evaluate associations between the trait response variables and candidate sets of environmental variables (see Table 2; **see****Supporting Information—Dataset S2**), we used the MuMIn (‘multimodel inference’) library in R to explore all combinations of the explanatory variables and rank the resulting models using the small-sample-corrected Akaike Information Criterion (AICc) (Bartoń 2016). We report the coefficients from the model with lowest AICc score (‘best model’) as well as this model’s *R*^2^ and the difference of its AICc score from the next-best-scoring model. To provide a more comprehensive picture of how strongly each explanatory variable is represented in the set of relatively high-scoring models—those within three AICc points of the best model—we also display the importance values of response variables included in this set of high-scoring models. A variable’s importance value is obtained by summing the AICc weights of the models in which it appears and calculating the ratio of this sum to the sum of the AICc weights for all models in the comparison set (Bartoń 2016).

**Table 2.** Candidate environmental variables and abbreviations. Variables used in assessing associations between *Erodium cicutarium* traits and source-site environment.

Variable name	Abbreviation	Type
Mean annual precipitation	MAP	Average climate conditions
Mean annual temperature	MAT	Average climate conditions
Interannual CV precipitation	PPTCV	Variation in climate
Precipitation seasonality	PPTSEAS	Variation in climate
Soil PC	Soil nut	Average soil conditions
CV of soil PC	CV soil nut	Variation in soil conditions
Total plant cover	Total cover	Average biotic conditions
CV total plant cover	CV total cover	Variation in biotic conditions

#### Trait correlations.

We calculated raw Pearson correlations among the trait means and the plasticity values to water and nutrient limitation, and displayed these visually using corrplot R library (Wei and Simko 2016). These values were calculated first for all families, then for each region (California or Chile) separately. Though we were mainly interested in the strength of these associations and their qualitative consistency among regions, we also tested for the significance of correlations using the confidence intervals of the Pearson correlations.

To test statistically whether these patterns of trait correlations varied by region, we used linear mixed models in a model comparison framework. For each pair of strongly correlated traits, we fitted a linear mixed model that included one of the two traits as the response variable, and the other trait, region, and interaction of trait and region as fixed effects, plus source population as a random effect. We compared these ‘full’ models using likelihood ratio tests to a ‘reduced’ model that omitted the effects of region and region-by-trait interaction.

## Results

### Genetic differentiation among populations and regions in mean trait expression and plasticity

**Figure 2.**
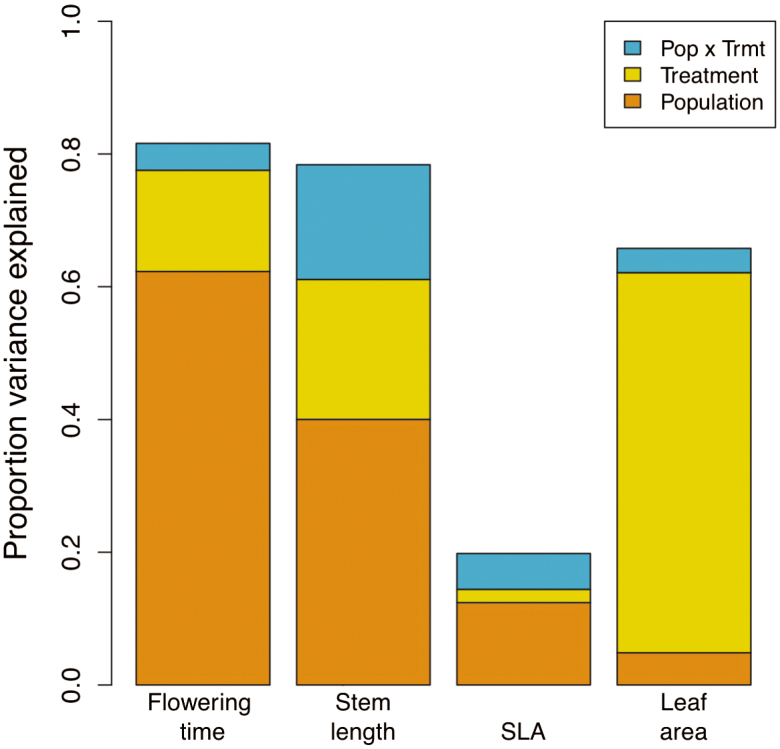
Decomposition of variance in trait means explained by experimental treatment, source population and their interaction. All effects were significant at the *P* < 0.01 level or less, except for the treatment × population interactions for SLA and leaf area, which were non-significant.

The mean values of flowering time, length of the longest stem, leaf area and SLA were significantly different both among treatments **[see****Supporting Information—Table S1****]** and among source populations (Table 3). In contrast, there were no significant differences in mean trait expression or plasticity between the two regions (Table 3). Overall, most of the variation in flowering time, stem length and leaf area could be explained by source population, treatment and their interaction, while most of the variation in SLA remained unexplained by these factors (Fig. 2). Source population explained the most variation in flowering time, stem length and SLA (Fig. 2; **see****Supporting Information—Table S2**). Most of the variation in leaf area, in contrast, was associated with plastic responses to treatments (smaller leaves in low-resource treatments) (Fig. 2; **see****Supporting Information—Table S2**).

**Table 3.** Tests of trait differences among regions and source populations. *P*-values from likelihood ratio tests were corrected for multiple comparisons for each set of 12 tests by controlling false discovery rate to 0.05.

Variable	Region		Population	
	χ^2^	*P*	χ^2^	*P*
Days to flower	0.54	0.55	880.1	<0.0001
log(Stem length)	0.00	0.99	432.3	<0.0001
log(SLA)	1.08	0.40	47.8	0.001
log(Leaf area)	0.02	0.97	361.8	<0.0001
Plasticity of flowering time to water	0.47	0.99	32.8	<0.0001
Plasticity of stem length to water	0.00	0.99	0.00	0.99
Plasticity of SLA to water	0.00	0.99	0.00	0.99
Plasticity of leaf area to water	0.00	0.99	0.34	0.99
Plasticity of flowering time to nutrients	0.00	0.99	0.00	0.99
Plasticity of stem length to nutrients	0.00	0.99	0.41	0.25
Plasticity of SLA to nutrients	0.00	0.99	1.26	0.99
Plasticity of leaf area to nutrients	0.00	0.99	0.00	0.99

**Figure 3.**
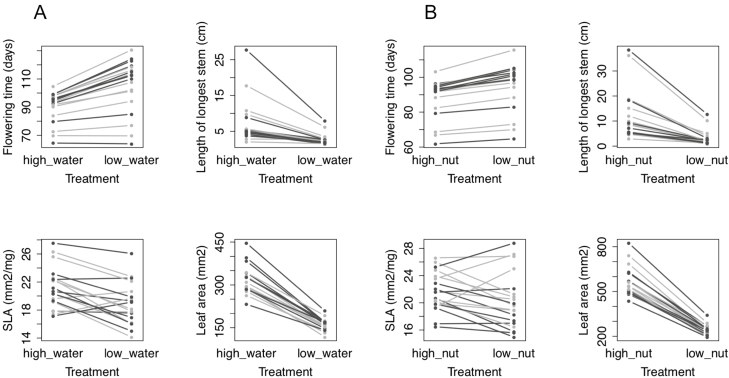
Reaction norms for *Erodium cicutarium* functional traits across treatments in a glasshouse experiment: (A) high- and low-water treatments; (B) high- and low-nutrient treatments. Populations from California are rendered in dark grey, and populations from Chile in light grey.

Plasticity in flowering time to contrasting water treatments was highly significantly different among populations (Table 3), but plasticity of flowering to nutrients was not (Table 3). Reaction norms to contrasting water and nutrient treatments (Fig. 3) show that trait values under all treatment levels were well-dispersed between the two regions. Despite significant differences in mean stem length, SLA and leaf area among source populations, there was no differentiation among source sites in the plasticity of these traits (Table 3).

With a few exceptions, families expressed either little plasticity in flowering time or delayed their flowering in response to low-water and low-nutrient treatments (Fig. 3). Similarly, families tended to express little plasticity in stem length or to grow shorter stems under resource limitation (Fig. 3). Families from all populations also tended to produce smaller leaves (lower leaf area) under both water and nutrient limitation (Fig. 3). Families therefore tended to flower later, at smaller size, and with smaller individual leaves under both water and nutrient limitation. Responses of SLA to water and nutrient limitation were qualitatively more heterogeneous among populations (Fig. 3), though as noted above, the levels of plasticity expressed among populations were not significantly different (Table 3).

### Associations between traits and source-population environmental factors

Associations between several trait means and environmental factors were identified using linear models. Trait variation among populations was most often associated with mean annual precipitation (Table 4). Plants from sites with higher precipitation flowered later, had shorter maximum stem length and had leaves with lower SLA (Table 4). In contrast with mean annual precipitation, mean annual temperature was a less important correlate of mean trait variation, with a moderate importance value for SLA and a low (<0.2) importance value for stem length and flowering time **[see****Supporting Information—Fig. S1a****]**. The soil principal component was associated only weakly with variation in trait means: the variable had a moderate to low importance value for flowering time, stem length and SLA **[see****Supporting Information—Fig. S1a****]**, and was included in best model only for SLA (Table 4). Mean total plant cover was not associated with trait means, and local variation in total cover had only one low importance value, for leaf area **[see****Supporting Information—Fig. S1a****]**. Similarly, local variation in soil properties (coefficient of variation of the soil principle component or PC) had a low importance value in models for SLA **[see****Supporting Information—Fig. S1a****]**.

**Table 4.** Trait–environment associations. Regression coefficients for environmental characteristics of source sites as predictors of trait expression of *Erodium cicutarium* plants grown in greenhouse treatments. Coefficients are reported for the single best model involving the set of predictors and their interaction with region, as ranked by model AICc value. For model evaluation, *R*^2^ and difference in AICc value from the second-ranked model are also reported.

Functional trait and environmental predictors	Coefficient	Standard error	*t*-value	*P*
Flowering time				
Mean annual precipitation	6.79	2.826	2.40	0.028
*R*^2^ = 0.25; ΔAICc = 1.45				
log(Stem length)				
Mean annual precipitation	−0.25	0.135	−1.84	0.083
*R*^2^ = 0.17; ΔAICc = 0.60				
log(SLA)				
Mean annual precipitation	−0.056	0.024	−2.33	0.033
Soil nutrients	−0.051	0.024	−2.10	0.052
*R*^2^ = 0.58; ΔAICc = 0.83				
log(Leaf area)				
Intercept-only model	NA	NA	NA	NA
*R*^2^ = NA; ΔAICc = 2.00				
Plasticity of flowering time to water				
Mean annual precipitation	0.040	0.012	−3.17	0.006
*R*^2^ = 0.37; ΔAICc = 1.49				
Plasticity of stem length to water				
Soil nutrients	−0.047	0.017	−2.79	0.013
*R*^2^ = 0.31; ΔAICc = 0.41				
Plasticity of SLA to water				
Intercept-only model	NA	NA	NA	NA
*R*^2^ = NA; ΔAICc = 2.19				
Plasticity of leaf area to water				
CV total plant cover	0.051	0.021	2.40	0.029
Total plant cover	0.053	0.021	2.46	0.026
*R*^2^ = 0.30; ΔAICc = 0.35				
Plasticity of flowering time to nutrients				
Mean annual precipitation	0.006	0.003	2.016	0.061
Total plant cover	−0.009	0.0029	−3.008	0.008
*R*^2^ = 0.40; ΔAICc = 1.04				
Plasticity of stem length to nutrients				
Mean annual temperature	0.036	0.13	2.69	0.016
Seasonality of precipitation	0.035	0.13	2.65	0.017
*R*^2^ = 0.33; ΔAICc = 0.82				
Plasticity of SLA to nutrients				
CV total plant cover	0.054	0.028	1.92	0.072
*R*^2^ = 0.18; ΔAICc = 0.87				
Plasticity of leaf area to nutrients				
Interannual CV precipitation	0.018	0.0094	1.92	0.072
*R*^2^ = 0.18; ΔAICc = 0.62				

Flowering time plasticity in response to variation in water availability was associated with mean annual precipitation: plants from wetter sites were more plastic in flowering time (**see****Supporting Information—Fig. S1b**; Table 4). Stem length plasticity to water, in contrast, was associated primarily with soil nutrients (mean of soil PC), and leaf area plasticity to water was associated with both mean total plant cover and variation in plant cover (**see****Supporting Information—Fig. S1b**; Table 4). As hypothesized, there were some associations between temporal variation in precipitation and trait plasticity to water availability, but these associations were weak and were never included in the best models (**see****Supporting Information—Fig. S1b**; Table 4).

Environmental associations with trait plasticity to nutrients were relatively different from the associations with plasticity to water. Flowering time plasticity to nutrients was most strongly associated with total plant cover, which appeared in all of the highly ranked models for this trait, and mean annual precipitation also had a high importance value (>0.75). Variation in stem length plasticity, in contrast, was associated most strongly with mean annual temperature and seasonality of precipitation (**see****Supporting Information—Fig. S1**; Table 4). Specific leaf area plasticity to nutrients was most strongly associated with variation in total plant cover, and leaf area plasticity was most strongly associated with interannual variation in precipitation (**see****Supporting Information—Fig. S1**; Table 4; Supporting Information—Fig. S2).

### Correlations among traits

**Figure 4.**
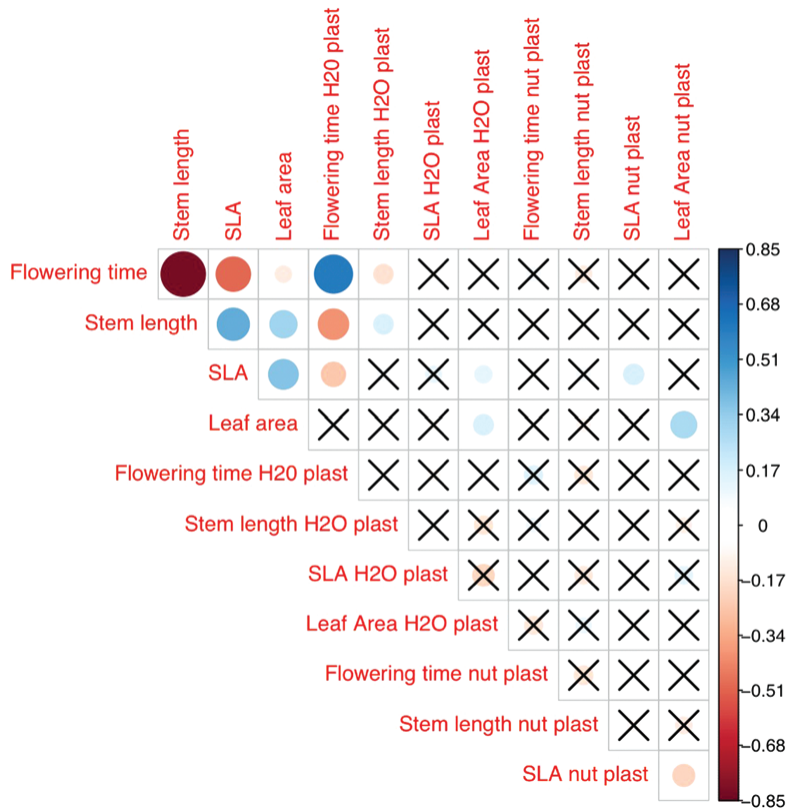
Correlations among traits and their plasticity for *Erodium cicutarium* maternal families grown in the greenhouse. Dot colour indicates the sign of correlations: red for negative correlations, blue for positive correlations. Dot size represents the absolute strength of correlations. Cells marked with ‘X’ contain correlations that are not statistically significant at the level *P* < 0.05. ‘H_2_O plast’ and ‘nut plast’ refer to trait plasticity to water and nutrients, respectively.

**Figure 5.**
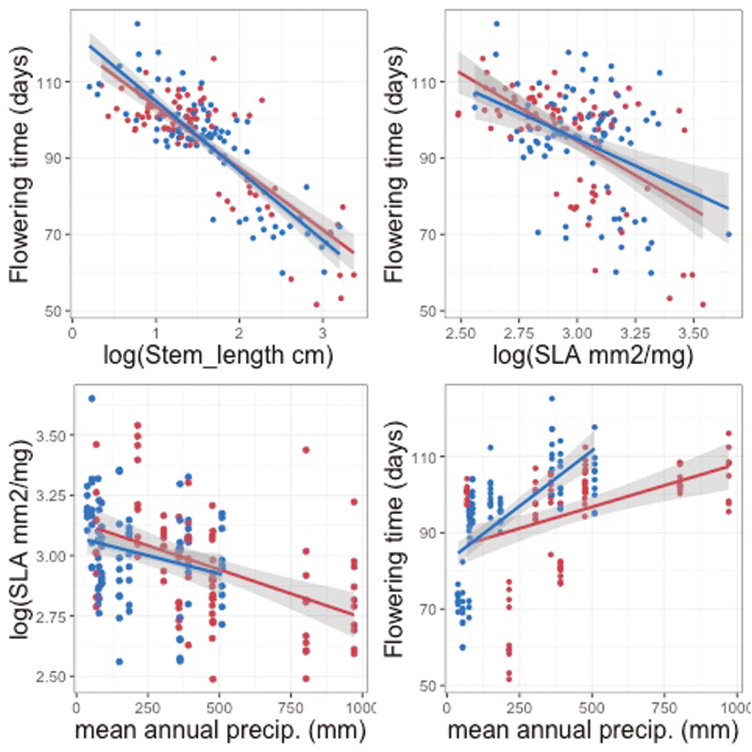
Trait–trait and trait–environment associations for *Erodium cicutarium* families from California and Chile. Red points represent means for California families, blue points represent means for Chilean families. Lines and associated shading display linear fits to the data with 95 % confidence intervals. Likelihood ratio tests indicated no interaction with region (*P* ≥ 0.18) (for the results of respective likelihood ratio tests, **see****Supporting Information—Table S4**), but for illustration, slopes are plotted separately by region: red lines represent linear fits for California, blue lines for Chile.

Mean plant size, estimated as length of the longest stem, and flowering time were correlated, so that families with fast growth rates and thus longer stems also tended to flower earlier (ρ = −0.58; *P* < 0.001; Fig. 4). Families that flowered later also tended to have lower SLA (ρ = −0.19; *P* < 0.001) and smaller leaves (ρ = −0.30; *P* < 0.001). There was also an association between stem length and both leaf area (ρ = 0.46; *P* < 0.001) and SLA (ρ = 0.13; *P* < 0.001). These correlation patterns were highly qualitatively consistent for the two regions. Figure 5 illustrates that the slopes of the relationships between flowering time and both SLA and plant size (length of longest stem) are essentially identical in the two regions, and that the relationship of SLA to mean annual precipitation is likewise consistent (Fig. 5). The relationship between flowering time and mean annual precipitation appears steeper (greater delay in flowering for the same increase in precipitation) in Chile, but the difference is not significant (*P* = 0.18; **see****Supporting Information—Table S3**). There were no significant interactions between any of these factors and region **[see Supporting Information—****Table S3****]**.

## Discussion

In this study of an exotic annual plant along parallel environmental gradients on two continents, we found strong evidence for genetic differentiation in response to environmental variation in the two invaded ranges. Despite the independent spread of the species in these two widely separated regions, there were no significant differences in either trait means or plasticity levels between the two regions. In contrast with the lack of divergence in traits *among* regions, there was a strong pattern of parallel divergence in functional traits *within* regions (Table 3; Fig. 4). Within both Chile and California, populations exhibited strong differentiation in mean traits and plasticity in flowering time, and this variation was associated with various environmental factors, most consistently with gradients in mean annual precipitation. Populations in dry areas on both continents appear to represent parallel evolution of an ecotype characterized by rapid growth during a short growing season, early flowering and relatively low plasticity in flowering time. Populations from wetter areas, in contrast, flower later and also express higher plasticity in flowering time under water limitation. Such strong, environmentally associated patterns in trait variation are consistent with patterns that have been found in other studies of invasive plants that have spread across climatic gradients (Maron *et al.* 2004; Colautti and Barrett 2013; Zenni *et al.* 2014). Previous research has shown that relatively rapid evolutionary differentiation is common in spreading invasive species; this study shows that for this invasive annual plant, the outcome of such differentiation is highly consistent in two invaded regions.

We observed a strong positive relationship between flowering time and mean annual precipitation: families of *E. cicutarium* collected from low precipitation sites in California and Chile flowered significantly earlier than families from high precipitation sites. Annual plants growing in Mediterranean regions worldwide generally emerge soon after the autumn rains begin and complete their life cycle before the onset of the dry, hot summer. Soil moisture levels drop to low levels earlier in the spring in sites with low average precipitation in such winter-rainfall climates, producing shorter growing seasons for plants. Short growing seasons are expected to favour rapid growth rates and early phenology in annual plants to ensure production of viable seeds before the onset of dry summers (Levitt 1980; Debieu *et al.* 2013; Donohue 2014), as also observed in desert regions where growth is strongly water-limited (Gremer *et al.* 2013). In arid sites, annual plants that delay flowering may not produce any viable seeds if flowering occurs too long after winter rains have ended. In contrast, in wetter sites, annuals that delay flowering may reach greater biomass before flowering, and this larger size may maximize their competitive ability and fitness. Consistent with these expectations, there is evidence that early flowering is often an adaptive response to drought in annuals (Heschel and Riginos 2005; Sherrard and Maherali 2006; Franks *et al.* 2007) and short-lived perennial forbs (Anderson *et al.* 2012).

Families collected from sites with low mean annual precipitation had leaves with higher SLA, on average, than maternal families from wetter sites. This negative relationship mirrors the effect of precipitation on average flowering time. Higher SLA in drier areas may seem counter-intuitive, given that lower SLA values are often associated with adaptation to tolerate dry climates, particularly in perennial plants (Stahl *et al.* 2013; Dwyer *et al.* 2014). However, *E. cicutarium* is an annual plant which adopts a drought-avoidance rather than a drought-tolerance strategy—it grows rapidly during the growth season, then senesces with the onset of drought (Kimball *et al.* 2014). Consistent with this ‘live fast, die young’ strategy (Donovan *et al.* 2007), it appears that this species is under selection to accelerate its life cycle in sites with lower annual precipitation, and that rapid growth enables it to maximize resource capture during the generally short growing season and rapidly produce seeds. Together, its traits form a correlated axis of variation associated with rapid growth (emergence to flowering in as little as 60 days), including high-SLA leaves that are relatively cheap to produce per unit area, need to be maintained only for a short time and can produce high growth rates (Galmes *et al*. 2005; Angert *et al.* 2007). The ecological strategy of drought avoidance is not limited to annual plants: for example, similar patterns in leaf morphology have been observed in other drought-avoiding plant species, such as drought-deciduous *Pelargonium* species from arid regions of South Africa (Jones *et al.* 2013).

Families from sites with higher precipitation exhibited greater plasticity in flowering time, which in this case means that they flowered later in response to both water and nutrient limitation than families from dry sites (Table 4). Higher plasticity in flowering time was only weakly associated with temporal variability in precipitation, both within and among years (**see****Supporting Information—Fig. S1b**; Table 4). Plasticity (i.e. greater delays) in flowering under water limitation was associated most strongly with mean annual precipitation and soil nutrient levels (**see****Supporting Information—Fig. S1**; Table 4). Early flowering time may be a trait in which reduced plasticity is favoured in sites with short growing seasons to maximize resource capture when water is available, and to ensure production of some seeds before the short growing season ends. For instance, another native annual forb from the Mediterranean-climate region of Chile shows null plasticity in flowering time to drought (Gonzales *et al.* 2016), even though it is fairly common across semiarid sites and exhibits significant reproductive outputs (Celedon-Neghme *et al.* 2007; Suarez *et al.* 2011). Lower plasticity in days to flowering in response to nutrient limitation (though not to water limitation) was also associated with higher total plant cover, a possible metric of competition intensity, in the source environment (**see****Supporting Information—Fig. S1c**; Table 4). A loss of plasticity in flowering time has also been found in *E. cicutarium* in response to shading in competition experiments, so it is possible there is a more general association between characteristics of biotic environment and flowering time plasticity (Vermeulen 2015; Heger 2016).

Trait plasticity in response to nutrient limitation was often associated with soil nutrient conditions in the source environment, but greater plasticity was not consistently associated with greater local variation in local nutrient availability. Similarly, mean nutrient levels were associated in inconsistent ways with levels of plasticity of different traits (**see****Supporting Information—Fig. S1b****and****c**; Table 4). These results are only weakly consistent with the hypothesis that higher plasticity will be associated with greater temporal variation in resource supply, here measured as the interannual coefficient of variation of precipitation. Interannual precipitation variation was weakly associated with plasticity in flowering in response to both water and nutrient availability, and with plasticity of stem length to nutrient availability, but not with plasticity of other traits in response to water (**see****Supporting Information—Fig. S1b****and****c**; Table 4). A recent review found a consistent pattern of greater plasticity in plant populations from sites with greater temporal heterogeneity in precipitation (Vazquez *et al*. 2017). However, in this review, the nine species that showed the expected association were all perennial plants, unlike *E. cicutarium*. This result could lead us to reconsider whether the expected positive association between plasticity and temporal variation should be as strong for short-lived plants.

This greenhouse experiment does not prove that the observed population differentiation in a suite of traits associated with an accelerated life cycle enhances fitness in the field at arid sites, since we have not planted the seeds reciprocally back into the source environments. Nonetheless, the consistency of the population differences and patterns of trait correlations across invaded ranges in both California and Chile suggests these patterns may be driven by selection rather than neutral evolutionary processes (Endler 1986). It is nonetheless possible, and has been found in some cases, that founder effects influence clinal variation in invaded ranges (Keller *et al.* 2009); we cannot rule such introduction effects out here, though the parallel patterns in the two regions suggest is less likely. The strong population differences in multiple correlated traits in two independent invasive ranges, coupled with potentially major fitness consequences of these traits, suggest that local adaptation to the tempo and length of the growing season has aided the spread of this species on both continents (Maron *et al.* 2007; Colautti and Barrett 2013). Despite the relatively short time the species has occupied these invaded ranges, it already displays strong patterns of differentiation qualitatively comparable to local differentiation in annual plant species in their native ranges. This evidence of substantial differentiation over a relatively short time period suggests that standing genetic variation is not necessarily low enough to strongly limit adaptation in small, colonizing plant populations, and that relatively rapid genetic differentiation in average trait values and phenotypic plasticity may play important roles in range expansion of invasive species. Our results provide, at a much shorter time scale, evidence that is consistent with the reported convergent evolution in form and function of vegetation from California and central Chile (Mooney 1977). From a different perspective, our results illustrate that invasive plant species may show similar adaptation patterns in comparable habitats, which can be of use for predicting their evolutionary trajectories in new invaded ranges.

## Supporting Information

The following additional information is available in the online version of this article—

Figure S1. Importance values for environmental explanatory variables.

Figure S2. Region-specific correlations among traits for *Erodium cicutarium* maternal families grown in the greenhouse.

Table S1. Results of tests for significance of treatment.

Table S2. Analysis of variance among populations and treatments corresponding to Fig. 2.

Table S3. Results of likelihood ratio tests for significant differences in trait associations between the two regions, Chile and California.

Dataset S1. *Erodium cicutarium* trait data by maternal family.

Dataset S2. Trait and environmental data for *Erodium cicutarium* source sites.

Supplementary_Dataset_S1

Supplementary_Dataset_S2

Supplementary_Tables_and_Figures

## Contributions by the Authors

B.S.J., A.M.L., and E.G. conceived of the study; B.S.J. and A.M.L. and C.S-L. collected seeds; B.S.J. and A.M.L. conducted the glasshouse experiment and analyzed the data; B.S.J. and A.M.L. wrote the paper with contributions from E.G., T.H., and C.S-L.

## Sources of Funding

This work was funded by NSF International Research Fellowship #0853094 (B.S.J.), by Hatch Project CA-D-PLS-2017-H (A.M.L.), and by Deutsche Forschungsgemeinschaft grants HE 5893/3-1 and HE 5893/3-2 and by Bavaria California Technology Center grant 9 [2001-1] (T.H.).

## Conflict of Interest

None declared.

## References

[CIT0001] Alleaume-Benharira M, Pen IR, Ronce O 2006 Geographical patterns of adaptation within a species’ range: interactions between drift and gene flow. Journal of Evolutionary Biology 19:203–215. 16405592 10.1111/j.1420-9101.2005.00976.x

[CIT0002] Alpert P, Simms EL 2002 The relative advantages of plasticity and fixity in different environments: when is it good for a plant to adjust? Evolutionary Ecology 16:285–297.

[CIT0003] Anderson JT, Inouye DW, McKinney AM, Colautti RI, Mitchell-Olds T 2012 Phenotypic plasticity and adaptive evolution contribute to advancing flowering phenology in response to climate change. Proceedings of the Royal Society B-Biological Sciences 279:3843–3852. 10.1098/rspb.2012.1051 PMC3415914 22787021

[CIT0004] Angert AL, Huxman TE, Barron-Gafford GA, Gerst KL, Venable DL 2007 Linking growth strategies to long-term population dynamics in a guild of desert annuals. Journal of Ecology 95:321–331.

[CIT0005] Balogh CM, Barrett SCH 2016 Stochastic processes during invasion: the influence of population size on style-morph frequency variation in *Lythrum salicaria* (purple loosestrife). International Journal of Plant Sciences 177:409–418.

[CIT0006] Barnéoud M 1845 Geranias. In: Gay C, ed. Historia Física y Política de Chile, Botánica. Paris: Fain et Thunot, 379–391.

[CIT0007] Bartoń K 2018 MuMIn: Multi-Model Inference. R package version 1.42.1. https://CRAN.R-project.org/package=MuMIn

[CIT0008] Baythavong BS 2011 Linking the spatial scale of environmental variation and the evolution of phenotypic plasticity: selection favors adaptive plasticity in fine-grained environments. The American Naturalist 178:75–87. 10.1086/660281 21670579

[CIT0009] Benjamini Y, Hochberg Y 1995 Controlling the false discovery rate - a practical and powerful approach to multiple testing. Journal of the Royal Statistical Society Series B-Methodological 57:289–300.

[CIT0010] Blossey B, Nuzzo V, Davalos A 2017 Climate and rapid local adaptation as drivers of germination and seed bank dynamics of *Alliaria petiolata* (garlic mustard) in North America. Journal of Ecology 105:1485–1495.

[CIT0011] Bossdorf O, Auge H, Lafuma L, Rogers WE, Siemann E, Prati D 2005 Phenotypic and genetic differentiation between native and introduced plant populations. Oecologia 144:1–11. 15891837 10.1007/s00442-005-0070-z

[CIT0012] Callaway RM, Pennings SC, Richards CL 2003 Phenotypic plasticity and interactions among plants. Ecology 84:1115–1128.

[CIT0013] Celedon-Neghme C, Gonzales WL, Gianoli E 2007 Cost and benefits of attractive floral traits in the annual species *Madia sativa* (Asteraceae). Evolutionary Ecology 21:247–257.

[CIT0014] Chevin LM, Lande R 2010 When do adaptive plasticity and genetic evolution prevent extinction of a density-regulated population? Evolution 64:1143–1150. 19863583 10.1111/j.1558-5646.2009.00875.x

[CIT0015] Chevin LM, Lande R, Mace GM 2010 Adaptation, plasticity, and extinction in a changing environment: towards a predictive theory. PLoS Biology 8 :e1000357. 10.1371/journal.pbio.1000357 PMC2864732 20463950

[CIT0016] Chown SL, Hodgins KA, Griffin PC, Oakeshott JG, Byrne M, Hoffmann AA 2015 Biological invasions, climate change and genomics. Evolutionary Applications 8:23–46. 25667601 10.1111/eva.12234 PMC4310580

[CIT0017] Colautti RI, Barrett SC 2013 Rapid adaptation to climate facilitates range expansion of an invasive plant. Science 342:364–366. 24136968 10.1126/science.1242121

[CIT0018] Colautti RI, Lau JA 2015 Contemporary evolution during invasion: evidence for differentiation, natural selection, and local adaptation. Molecular Ecology 24:1999–2017. 25891044 10.1111/mec.13162

[CIT0019] Debieu AM, Jennions M, Nicotra AB 2011 Do invasive species show higher phenotypic plasticity than native species and, if so, is it adaptive? A meta-analysis. Ecology Letters 14:419–431. 21314880 10.1111/j.1461-0248.2011.01596.x

[CIT0020] Debieu M, Tang C, Stich B, Sikosek T, Effgen S, Josephs E, Schmitt J., Nordborg M., Koornneef M, de Meaux J 2013 Co-variation between seed dormancy, growth rate and flowering time changes with latitude in *Arabidopsis thaliana*. PLoS One 8:e61075. 10.1371/journal.pone.0061075 PMC3662791 23717385

[CIT0021] Di Castri F 1973 Climatographical comparisons between Chile and the western coast of North America. In: Di Castri F, ed. Mediterranean type ecosystems: origin and structure. Berlin: Springer-Verlag, 21–36.

[CIT0022] Donohue K 2014 Why ontogeny matters during adaptation: developmental niche construction and pleiotorpy across the life cycle in *Arabidopsis thaliana*. Evolution 68:32–47. 24117399 10.1111/evo.12284

[CIT0023] Donovan LA, Dudley SA, Rosenthal DM, Ludwig F 2007 Phenotypic selection on leaf water use efficiency and related ecophysiological traits for natural populations of desert sunflowers. Oecologia 152:13–25. 17165094 10.1007/s00442-006-0627-5

[CIT0024] Dwyer JM, Hobbs RJ, Mayfield MM 2014 Specific leaf area responses to environmental gradients through space and time. Ecology 95:399–410. 24669733 10.1890/13-0412.1

[CIT0025] Endler JA 1986 Natural Selection in the wild. Princeton, NJ: Princeton University Press.

[CIT0026] Fiz-Palacios O, Vargas P, Vila R, Papadopulos AS, Aldasoro JJ 2010 The uneven phylogeny and biogeography of *Erodium* (Geraniaceae): radiations in the Mediterranean and recent recurrent intercontinental colonization. Annals of Botany 106:871–884. 20858592 10.1093/aob/mcq184 PMC2990656

[CIT0027] Fournier-Level A, Korte A, Cooper MD, Nordborg M, Schmitt J, Wilczek AM 2011 A map of local adaptation in *Arabidopsis thaliana*. Science 334:86–89. 21980109 10.1126/science.1209271

[CIT0028] Franks SJ, Sim S, Weis AE 2007 Rapid evolution of flowering time by an annual plant in response to a climate fluctuation. Proceedings of the National Academy of Sciences of the United States of America 104:1278–1282. 17220273 10.1073/pnas.0608379104 PMC1783115

[CIT0029] Franks SJ, Weber JJ, Aitken SN 2014 Evolutionary and plastic responses to climate change in terrestrial plant populations. Evolutionary Applications 7:123–139. 24454552 10.1111/eva.12112 PMC3894902

[CIT0030] Galmes J, Cifre J, Medrano H, Flexas J 2005 Modulation of relative growth rate and its components by water stress in Mediterranean species with different growth forms. Oecologia 145:21–31. 15868165 10.1007/s00442-005-0106-4

[CIT0031] Gianoli E 2003 Phenotypic responses of the twining vine *Ipomoea purpurea* (Convolvulaceae) to physical support availability in sun and shade. Plant Ecology 165:21–26.

[CIT0032] Gianoli E 2004 Plasticity of traits and correlations in two populations of *Convolvulus arvensis* (Convolvulaceae) differing in environmental heterogeneity. International Journal of Plant Sciences 165:825–832.

[CIT0033] Gianoli E, Gonzalez-Teuber M 2005 Environmental heterogeneity and population differentiation in plasticity to drought in *Convolvulus chilensis* (Convolvulaceae). Evolutionary Ecology 19:603–613.

[CIT0034] Gonzales WL, Suarez LH, Gianoli E 2016 Genetic variation in the reduction of attractive floral traits of an annual tarweed in response to drought and apical damage. Journal of Plant Ecology 9:629–635.

[CIT0035] Gremer JR, Kimball S, Keck KR, Huxman TE, Angert AL, Venable DL 2013 Water-use efficiency and relative growth rate mediate competitive interactions in Sonoran Desert winter annual plants. American Journal of Botany 100:2009–2015. 24095798 10.3732/ajb.1300064

[CIT0036] Heger T 2016 Light availability experienced in the field affects ability of following generations to respond to shading in an annual grassland plant. Journal of Ecology 104:1432–1440.

[CIT0037] Hereford J 2009 A quantitative survey of local adaptation and fitness trade-offs. The American Naturalist 173:579–588. 10.1086/597611 19272016

[CIT0038] Heschel MS, Riginos C 2005 Mechanisms of selection for drought stress tolerance and avoidance in *Impatiens capensis* (Balsaminaceae). American Journal of Botany 92:37–44. 21652382 10.3732/ajb.92.1.37

[CIT0039] Hobbs RJ, Valentine LE, Standish RJ, Jackson ST 2018 Movers and stayers: novel assemblages in changing environments. Trends in Ecology & Evolution 33:116–128. 29173900 10.1016/j.tree.2017.11.001

[CIT0040] Holt RD, Barfield M 2011 Theoretical perspectives on the statics and dynamics of species’ borders in patchy environments. The American Naturalist 178:S6–S25. 10.1086/661784 PMC5014989 21956092

[CIT0040a] Hulme, M. (1992) A 1951-80 global land precipitation climatology for the evaluation of general circulation models. Climate Dynamics 7:57–72.

[CIT0041] Jones FC, Grabherr MG, Chan YF, Russell P, Mauceli E, Johnson J, Swofford R, Pirun M, Zody MC, White S, Birney E, Searle S, Schmutz J, Grimwood J, Dickson MC, Myers RM, Miller CT, Summers BR, Knecht AK, Brady SD, Zhang H, Pollen AA, Howes T, Amemiya C, Baldwin J, Bloom T, Jaffe DB, Nicol R, Wilkinson J, Lander ES, Di Palma F, Lindblad-Toh K, Kingsley DM; Broad Institute Genome Sequencing Platform & Whole Genome Assembly Team 2012 The genomic basis of adaptive evolution in threespine sticklebacks. Nature 484:55–61. 22481358 10.1038/nature10944 PMC3322419

[CIT0042] Jones CS, Martínez-Cabrera HI, Nicotra AB, Mocko K, Marais EM, Schlichting CD 2013 Phylogenetic influences on leaf trait integration in *Pelargonium* (Geraniaceae): convergence, divergence, and historical adaptation to a rapidly changing climate. American Journal of Botany 100:1306–1321. 23825139 10.3732/ajb.1200526

[CIT0043] Keller SR, Sowell DR, Neiman M, Wolfe LM, Taylor DR 2009 Adaptation and colonization history affect the evolution of clines in two introduced species. The New Phytologist 183:678–690. 19538550 10.1111/j.1469-8137.2009.02892.x

[CIT0044] Kimball S, Gremer JR, Barron-Gafford GA, Angert AL, Huxman TE, Venable DL 2014 High water-use efficiency and growth contribute to success of non-native *Erodium cicutarium* in a Sonoran Desert winter annual community. Conservation Physiology 2:cou006. 10.1093/conphys/cou006 PMC4806723 27293627

[CIT0045] Lazaro-Nogal A, Matesanz S, Godoy A, Perez-Trautman F, Gianoli E, Valladares F 2015 Environmental heterogeneity leads to higher plasticity in dry-edge populations of a semi-arid Chilean shrub: insights into climate change responses. Journal of Ecology 103:338–350.

[CIT0046] Leger EA, Rice KJ 2007 Assessing the speed and predictability of local adaptation in invasive California poppies (*Eschscholzia californica*). Journal of Evolutionary Biology 20:1090–1103. 17465919 10.1111/j.1420-9101.2006.01292.x

[CIT0047] Leimu R, Fischer M 2008 A meta-analysis of local adaptation in plants. PLoS One 3:e4010. 10.1371/journal.pone.0004010 PMC2602971 19104660

[CIT0048] Levitt J 1980 Responses of plants to environmental stresses. New York: Academic Press.

[CIT0049] Linhart YB, Grant MC 1996 Evolutionary significance of local genetic differentiation in plants. Annual Review of Ecology and Systematics 27:237–277.

[CIT0050] Maron JL, Elmendorf SC, Vilà M 2007 Contrasting plant physiological adaptation to climate in the native and introduced range of *Hypericum perforatum*. Evolution 61:1912–1924. 17683433 10.1111/j.1558-5646.2007.00153.x

[CIT0051] Maron JL, Vila M, Bommarco R, Elmendorf S, Beardsley P 2004 Rapid evolution of an invasive plant. Ecological Monographs 74:261–280.

[CIT0052] Matesanz S, Horgan-Kobelski T, Sultan SE 2014 Contrasting levels of evolutionary potential in populations of the invasive plant *Polygonum cespitosum*. Biological Invasions 16:455–468.

[CIT0053] Mensing S, Byrne R 1998 Pre-mission invasion of *Erodium cicutarium* in California. Journal of Biogeography 25:757–762.

[CIT0054] Molina-Montenegro MA, del Pozo A, Gianoli E 2018 Ecophysiological basis of the Jack-and-Master strategy: *Taraxacum officinale* (Dandelion) as an example of a successful invader. Journal of Plant Ecology 11:147–157.

[CIT0055] Molina-Montenegro MA, Palma-Rojas C, Alcayaga-Olivares Y, Oses R, Corcuera LJ, Cavieres LA, Gianoli E 2013 Ecophysiological plasticity and local differentiation help explain the invasion success of *Taraxacum officinale* (Dandelion) in South America. Ecography 36:718–730.

[CIT0056] Montague JL, Barrett SCH, Eckert CG 2008 Re-establishment of clinal variation in flowering time among introduced populations of purple loosestrife (*Lythrum salicaria*, Lythraceae). Journal of Evolutionary Biology 21:234–245. 18028354 10.1111/j.1420-9101.2007.01456.x

[CIT0057] Mooney HA 1977 Convergent evolution in Chile and California: Mediterranean climate ecosystems. Stroudsburg, PA; [New York]: Dowden, Hutchinson & Ross; exclusive distributor Halsted Press.

[CIT0058] Moran EV, Alexander JM 2014 Evolutionary responses to global change: lessons from invasive species. Ecology Letters 17:637–649. 24612028 10.1111/ele.12262

[CIT0059] Moran EV, Reid A, Levine JM 2017 Population genetics and adaptation to climate along elevation gradients in invasive *Solidago canadensis*. PLoS One 12: e0185539 10.1371/journal.pone.0185539 PMC5619793 28957402

[CIT0060] Murren CJ, Dudash MR 2012 Variation in inbreeding depression and plasticity across native and non-native field environments. Annals of Botany 109:621–632. 22247124 10.1093/aob/mcr325 PMC3278298

[CIT0061] Oduor AMO, Leimu R, van Kleunen M 2016 Invasive plant species are locally adapted just as frequently and at least as strongly as native plant species. Journal of Ecology 104:957–968.

[CIT0062] Parker IM, Rodriguez J, Loik ME 2003 An evolutionary approach to understanding the biology of invasions: local adaptation and general-purpose genotypes in the weed *Verbascum thapsus*. Conservation Biology 17:59–72.

[CIT0063] Pigliucci M, Schlichting CD 1995 Ontogenetic reaction norms in *Lobelia siphilitica* (Lobeliaceae): response to shading. Ecology 76:2134–2144.

[CIT0064] Pratt JD, Mooney KA 2013 Clinal adaptation and adaptive plasticity in *Artemisia californica*: implications for the response of a foundation species to predicted climate change. Global Change Biology 19:2454–2466. 23505064 10.1111/gcb.12199

[CIT0065] Prentis PJ, Wilson JR, Dormontt EE, Richardson DM, Lowe AJ 2008 Adaptive evolution in invasive species. Trends in Plant Science 13:288–294. 18467157 10.1016/j.tplants.2008.03.004

[CIT0066] R Core Team 2018 R: a language and environment for statistical computing. Vienna, Austria: R Foundation for Statistical Computing.

[CIT0067] Richards CL, Bossdorf O, Muth NZ, Gurevitch J, Pigliucci M 2006 Jack of all trades, master of some? On the role of phenotypic plasticity in plant invasions. Ecology Letters 9:981–993. 16913942 10.1111/j.1461-0248.2006.00950.x

[CIT0068] Richardson DM, Pyšek P, Rejmánek M, Barbour MG, Panetta FD, West CJ 2000 Naturalization and invasion of alien plants: concepts and definitions. Diversity and Distributions 6:93–107.

[CIT0069] Savage JA, Cavender-Bares JM 2011 Contrasting drought survival strategies of sympatric willows (genus: *Salix*): consequences for coexistence and habitat specialization. Tree Physiology 31:604–614. 21778293 10.1093/treephys/tpr056

[CIT0070] Sexton JP, McKay JK, Sala A 2002 Plasticity and genetic diversity may allow saltcedar to invade cold climates in North America. Ecological Applications 12:1652–1660.

[CIT0071] Sherrard ME, Maherali H 2006 The adaptive significance of drought escape in *Avena barbata*, an annual grass. Evolution 60:2478–2489. 17263110

[CIT0072] Stahl U, Kattge J, Reu B, Voigt W, Ogle K, Dickie J, Wirth C 2013 Whole-plant trait spectra of North American woody plant species reflect fundamental ecological strategies. Ecosphere 4:1–28.

[CIT0073] Suarez LH, Perez F, Armesto JJ 2011 Strong phenotypic variation in floral design and display traits of an annual tarweed in relation to small-scale topographic heterogeneity in semiarid Chile. International Journal of Plant Sciences 172:1012–1025.

[CIT0074] Sultan SE, Spencer HG 2002 Metapopulation structure favors plasticity over local adaptation. The American Naturalist 160:271–283. 10.1086/341015 18707492

[CIT0075] Van Kleunen M, Fischer M 2001 Adaptive evolution of plastic foraging responses in a clonal plant. Ecology 82:3309–3319.

[CIT0076] Vázquez DP, Gianoli E, Morris WF, Bozinovic F 2017 Ecological and evolutionary impacts of changing climatic variability. Biological Reviews of the Cambridge Philosophical Society 92:22–42. 26290132 10.1111/brv.12216

[CIT0077] Vermeulen PJ 2015 On selection for flowering time plasticity in response to density. The New Phytologist 205:429–439. 25124368 10.1111/nph.12984

[CIT0078] Weber E, Schmid B 1998 Latitudinal population differentiation in two species of *Solidago* (Asteraceae) introduced into Europe. American Journal of Botany 85:1110. 21684996

[CIT0079] Wei T, Simko V 2017 corrplot: Visualization of a Correlation Matrix. R Package version 0.84. https://github.com/taiyun/corrplot

[CIT0080] Zenni RD, Bailey JK, Simberloff D 2014 Rapid evolution and range expansion of an invasive plant are driven by provenance-environment interactions. Ecology Letters 17:727–735. 24703489 10.1111/ele.12278

