# Supplementary material for: Parallel functional differentiation of an invasive annual plant on two continents
Source: AoB Plants. 2019 Mar 7;11(2):plz010. doi: 10.1093/aobpla/plz010 (PMC6479022; doi:10.1093/aobpla/plz010)
Supplement: Supplementary_Tables_and_Figures [file plz010_suppl_supplementary_tables_and_figures.pdf]

Supplementary Tables

Table S1. Results of tests for significance of treatment. P-values were corrected for multiple comparisons by controlling false discovery rate to 0.05.

Variable	F	P
Flowering time	164.6	<0.0001
log(Stem length)	129.3	<0.0001
log(SLA)	5.6	0.0013
log(Leaf area)	361.8	<0.0001

Table S2. Analysis of variance among populations and treatments corresponding to Figure 2.

**Flowering time**					
**Variable**	**df**	**SS**	**SSmean**	**F**	**P**
Population	18	128834	7157.4	125.97	<0.0001
Treatment	3	31584	185.3	185.29	<0.0001
Population:Treatment	54	8435	156.2	2.75	<0.0001
Residuals	669	38013	56.8		

**log(stem length)**					
**Variable**	**df**	**SS**	**SSmean**	**F**	**P**
Population	18	250.7	13.9	52.32	<0.0001
Treatment	3	239.7	79.9	300.19	<0.0001
Population:Treatment	54	23.1	0.4	1.60	0.0050
Residuals	660	175.7	0.3		

**log(SLA)**					
**Variable**	**df**	**SS**	**SSmean**	**F**	**P**
Population	18	7.05	0.29	3.56	<0.0001
Treatment	3	6.05	2.02	18.31	<0.0001
Population:Treatment	54	5.35	0.10	0.90	0.68
Residuals	588	64.77	0.11		

**Leaf area**					
**Variable**	**df**	**SS**	**SSmean**	**F**	**P**
Population	18	9.1	0.51	4.72	<0.0001
Treatment	3	147.3	49.10	457.20	<0.0001
Population:Treatment	54	6.3	0.12	1.08	0.32
Residuals	660	70.9	0.11		

Table S3. Results of Likelihood ratio tests for significant differences in trait associations between the two regions, Chile and California. The test statistic and p-value represents a test of whether including region and region-by-trait interaction improves a linear mixed model for the association between two functional traits, or between a functional trait and an environmental variable.

Trait association	Likelihood ratio test Chi-square value	df	P
Days to first flowering vs. stem length	2.93	2	0.23
Days to first flowering vs. SLA	3.31	2	0.19
log(SLA) vs. mean annual precipitation	0.92	2	0.63
Days to first flowering vs. mean annual precipitation	3.45	2	0.18

Supplementary Figures

Figure S1. Importance values for environmental explanatory variables. Bars represent importance values of variables in highly ranked linear models that relate trait values to source-site environmental variables. The y-axis represents the importance value of the variable across a set of highly-ranked models: those with small-sample-corrected AIC scores (AICc) within 3 points of the highest-ranked model. The importance value is proportional to the sum of AICc scores for the models in which the variable appears.

a) Trait means

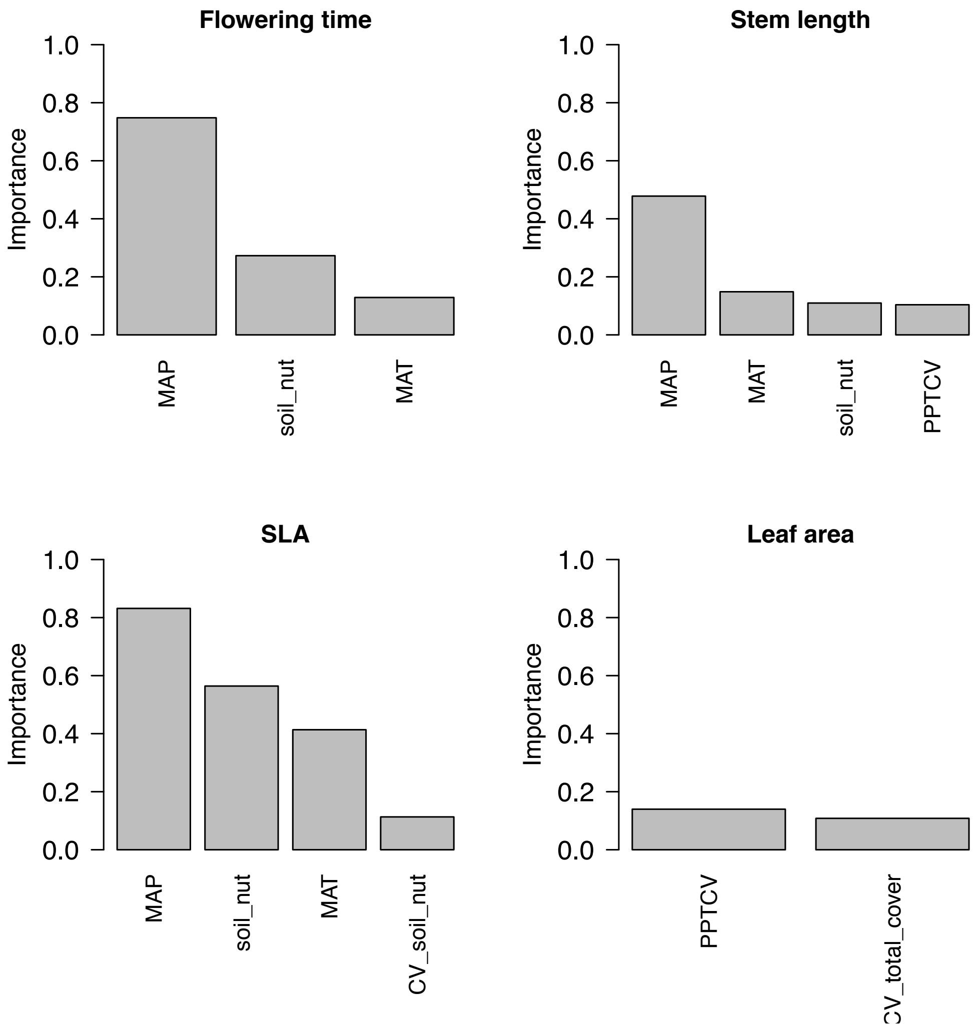

b) Trait plasticity to variation in water availability

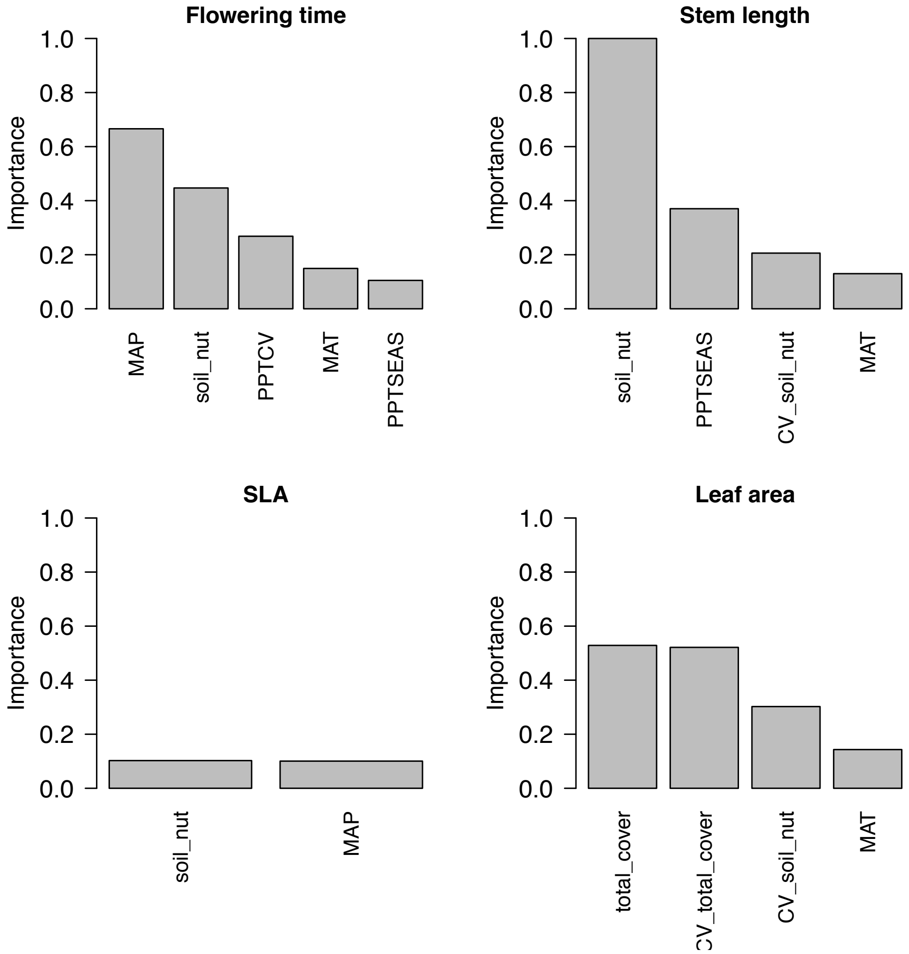

c) Trait plasticity to variation in nutrient availability

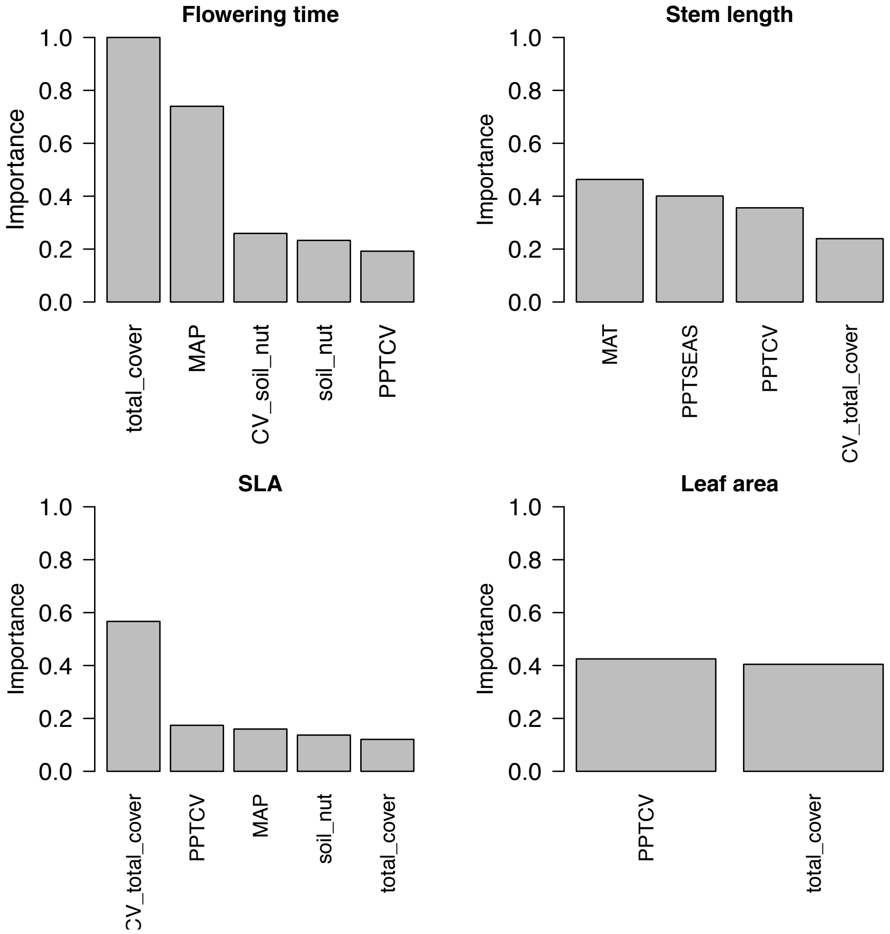

Figure S2. Region-specific correlations among traits for *Erodium cicutarium* maternal families grown in the greenhouse. Here the correlation patterns are shown separately by source region: a) California and b) Chile. Plasticity values displayed here are the plasticity of traits in response to variation in water availability. Colors indicate the direction and strength of correlations, with red indicating negative correlations and blue indicating positive, as indicated on the scale bars. The size of the circles represent their absolute strength. Cells marked with "X" contain correlations that are not statistically significant at the level $P < 0.05$.

(a)

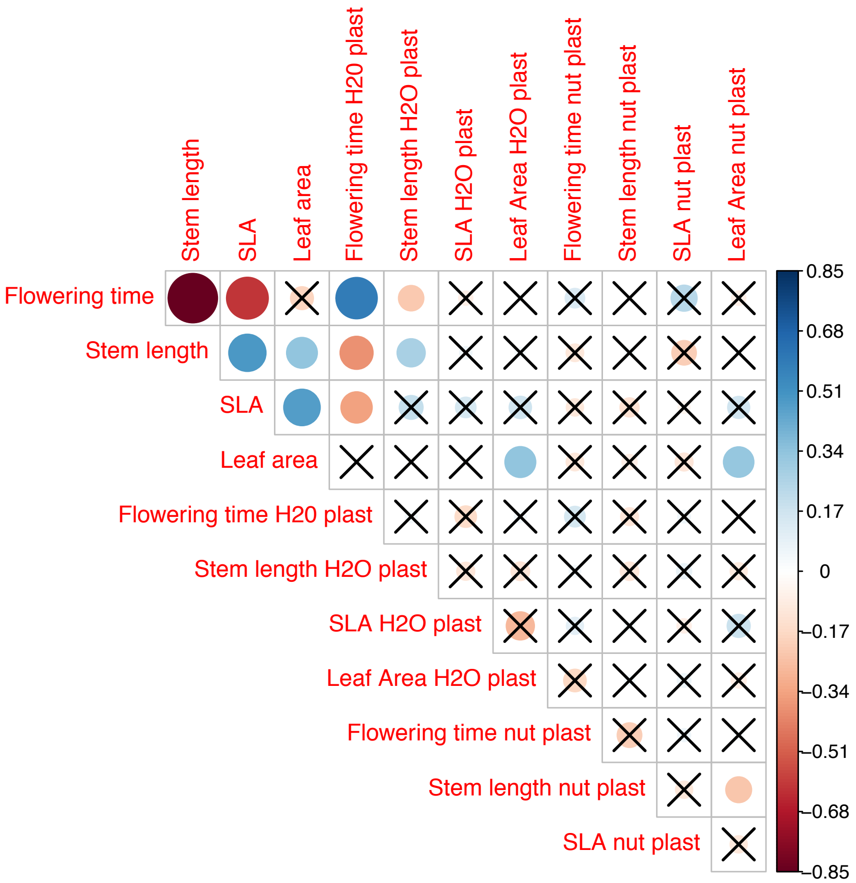

(b)

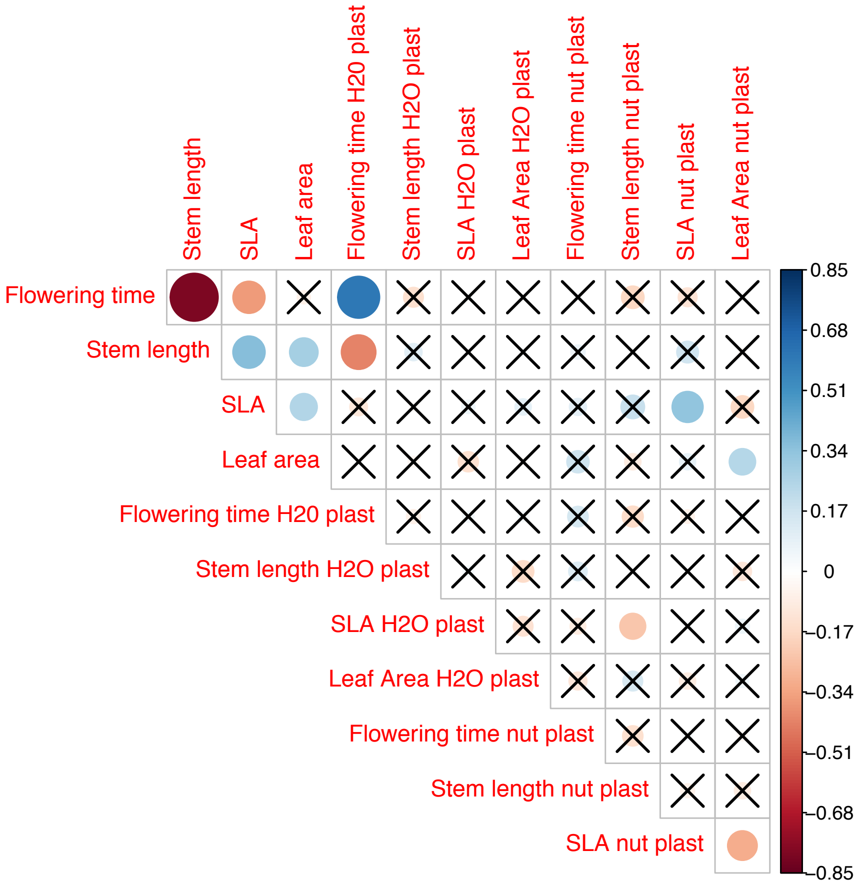

Stem length
SLA
Leaf area
Flowering time H20 plast
Stem length H2O plast
SLA H2O plast
Leaf Area H2O plast
Flowering time nut plast
Stem length nut plast
SLA nut plast
Leaf Area nut plast
Flowering time
Stem length
SLA
Leaf area
Flowering time H20 plast
Stem length H2O plast
SLA H2O plast
Leaf Area H2O plast
Flowering time nut plast
Stem length nut plast
SLA nut plast
0.85
0.68
0.51
0.34
0.17
0
-0.17
-0.34
-0.51
-0.68
-0.85
